# A Cost-Effectiveness Model for Adjunctive Smoked Cannabis in the Treatment of Chronic Neuropathic Pain

**DOI:** 10.1089/can.2018.0027

**Published:** 2019-03-13

**Authors:** Griffin A. Tyree, Reith Sarkar, Brandon K. Bellows, Ronald J. Ellis, Joseph Hampton Atkinson, Thomas D. Marcotte, Mark S. Wallace, Igor Grant, Yuyan Shi, James D. Murphy, David J. Grelotti

**Affiliations:** ^1^ School of Medicine, University of California San Diego, La Jolla, California.; ^2^ Division of General Medicine, Columbia University, New York, New York.; ^3^ Department of Psychiatry, University of California San Diego, La Jolla, California.; ^4^ University of California Center for Medicinal Cannabis Research, San Diego, California.; ^5^ Department of Neurosciences, University of California San Diego, La Jolla, California.; ^6^ Department of Anesthesiology, University of California San Diego, La Jolla, California.; ^7^ Department of Family Medicine and Public Health, University of California San Diego, La Jolla, California.; ^8^ Department of Radiation Medicine and Applied Science, University of California San Diego, La Jolla, California.

**Keywords:** cost-effectiveness, medical marijuana, painful neuropathy, diabetic neuropathy, HIV neuropathy

## Abstract

**Background:** A recent meta-analysis affirmed the benefit of medicinal cannabis for chronic neuropathic pain, a disabling and difficult-to-treat condition. As medicinal cannabis use is becoming increasingly prevalent among Americans, an exploration of its economic feasibility is warranted. We present this cost-effectiveness analysis of adjunctive cannabis pharmacotherapy for chronic peripheral neuropathy.

**Materials and Methods:** A published Markov model comparing conventional therapies for painful diabetic neuropathy was modified to include arms for augmenting first-line, second-line (if first-line failed), or third-line (if first- and second-line failed) therapies with smoked cannabis. Microsimulation of 1,000,000 patients compared the cost (2017 U.S. dollars) and effectiveness (quality-adjusted life years [QALYs]) of usual care with and without adjunctive cannabis using a composite of third-party and out-of-pocket costs. Model efficacy inputs for cannabis were adapted from clinical trial data. Adverse event rates were derived from a prospective study of cannabis for chronic noncancer pain and applied to probability inputs for conventional therapies. Cannabis cost was derived from retail market pricing. Parameter uncertainty was addressed with one-way and probabilistic sensitivity analysis.

**Results:** Adding cannabis to first-line therapy was incrementally less effective and costlier than adding cannabis to second-line and third-line therapies. Third-line adjunctive cannabis was subject to extended dominance, that is, the second-line strategy was more effective with a more favorable incremental cost-effectiveness ratio of $48,594 per QALY gained, and therefore, third-line adjunctive cannabis was not as cost-effective. At a modest willingness-to-pay threshold of $100,000/QALY gained, second-line adjunctive cannabis was the strategy most likely to be cost-effective.

**Conclusion:** As recently proposed willingness-to-pay thresholds for the United States health marketplace range from $110,000 to $300,000 per QALY, cannabis appears cost-effective when augmenting second-line treatment for painful neuropathy. Further research is warranted to explore the long-term benefit of smoked cannabis and standardization of its dosing for chronic neuropathic pain.

## Introduction

A growing body of scientific literature demonstrates reproducible efficacy of cannabis in the treatment of several medical conditions, including chronic neuropathic pain. Clinical trials of oral,^1–5^ smoked,^6–9^ and vaporized^10,11^ cannabis and cannabinoids have all demonstrated analgesic benefit of medicinal cannabis in the treatment of this costly^12^ and disabling^13,14^ condition. A recent meta-analysis of individual patient data from five randomized controlled trials of inhaled cannabis demonstrated pain relief comparable to gabapentin.^15^ Treatment guidelines for neuropathic pain recommend consideration of cannabinoids as third-line agents.^16^

An increasing number of patients are using cannabis for medical reasons,^17–23^ but how do we know if the health benefits gained with medicinal cannabis are worth the added cost? Cost-effectiveness analysis (CEA) compares the costs and health benefits of two or more interventions to determine their value. A treatment is considered cost-effective when the ratio of incremental costs to incremental health benefits, known as the incremental cost-effectiveness ratio (ICER),^24^ is less than a health care payer's willingness to pay for the health benefit. Quality-adjusted life years (QALYs), which incorporate both quality of life and longevity, are the recommended measure of health benefit.^24,25^

Conversely, treatment is considered dominated—and categorically not cost-effective—when it is less effective but more costly than an alternative, and extendedly dominated when there is another treatment alternative to usual care with a lower ICER value. In the United States, $50,000 per QALY is a commonly accepted willingness-to-pay threshold, but expert opinion estimates that it likely ranges from $110,000 to $300,000 per QALY.^26–28^

To our knowledge, the only cost-effectiveness studies of cannabis or cannabinoids evaluated nabiximols and dronabinol for multiple sclerosis (MS),^29^ and no studies have assessed the cost-effectiveness of smoked medicinal cannabis for any condition. As federal regulations prohibit health plans from covering medicinal cannabis and patient expenses are out-of-pocket, knowing the cost-effectiveness of medicinal cannabis may impact how providers advise its use in patients suffering from chronic neuropathic pain.

As the majority of patients in placebo-controlled trials of medicinal cannabis were administered cannabis in addition to an existing pain regimen,^6–8,11^ there is sufficient data to support an exploratory study of the cost-effectiveness of smoked cannabis as adjunctive therapy in the treatment of chronic neuropathic pain. The purpose of this exploratory computer simulation study was to assess the cost-effectiveness of augmenting first-line, second-line, or third-line standard therapies for neuropathic pain with smoked cannabis in treatment-naive patients over 1 year from a U.S. health care sector perspective. We also sought to assess the robustness of our simulation to changes in parameter inputs and assumptions.^30^

## Materials and Methods

### Overview

CEAs often use branching decision models constructed in specialized software to compare interventions in simulated patients. In CEA models, simulated patients experience clinical outcomes related to health state utility and decrements (e.g., clinical improvement, intolerable side effects, or death) and incur costs (e.g., prescription medication fills, physician office visits, or hospitalizations) based on input probabilities, which are commonly derived from published literature.^30^

For this analysis, we constructed a CEA model by adding adjunctive cannabis to the treatment arms of a published microsimulation (i.e., individual-patient simulation) model of painful diabetic peripheral neuropathy (pDPN).^31^ While the efficacy of smoked cannabis from clinical trials represents a heterogeneous group of conditions causing neuropathic pain, we did not identify a published microsimulation model for chronic neuropathic pain due to mixed etiologies. Although multiple published CEAs of chronic neuropathic pain due to a single etiology were identified,^31–34^ the model by Bellows et al. best approximated clinical practice by allowing patients to switch between standard therapy agents when they experienced poor pain relief or adverse events.^31^

We therefore adapted this model to estimate the costs (2017 U.S. dollars), QALYs, and cost-effectiveness of augmenting standard therapy agents for neuropathic pain with smoked cannabis by adding parameters to simulate adjunctive cannabis use.

### Model structure

We simulated the cost and QALY outcomes of 1,000,000 treatment-naive patients newly diagnosed with neuropathic pain. Following Bellows et al., baseline age and pain score were assigned from normal distributions derived from pooled, weighted means and standard deviations from clinical trials of four standard therapy agents for neuropathic pain (i.e., desipramine, duloxetine, gabapentin, and pregabalin).^31^ As the previous analysis found that initiating duloxetine in treatment-naive patients with pDPN was the most cost-effective first-line therapy,^31^ we assumed that each patient would receive standard therapy beginning with duloxetine.

In the event of drug failure, patients then switched randomly to one of three remaining standard therapy agents. In our model, adjunctive cannabis was initiated according to four treatment strategies: (1) never (the “usual care” arm), (2) at the start of treatment (first-line adjunctive cannabis), (3) after failing one standard therapy agent (second-line adjunctive cannabis), or (4) after failing two standard therapy agents (third-line adjunctive cannabis).

**FIG. 1.**
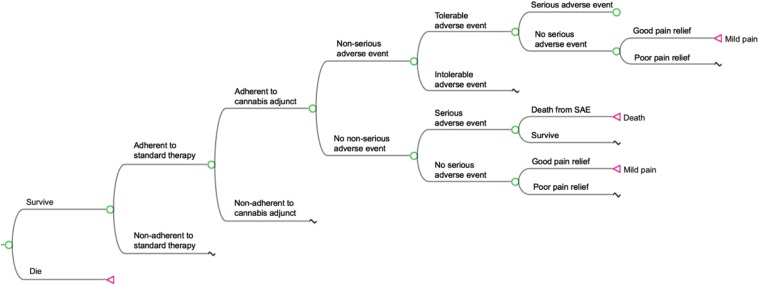
Abbreviated model overview. Pictured is a node structure wherein adjunctive cannabis is integrated into a treatment model using standard therapy agents described by Bellows et al.^31^ Beginning in a moderate-to-severe pain health state, simulated patients are assessed stepwise for mortality, adherence, tolerable or intolerable adverse events, SAE, and quality of pain relief. Patients who die are removed from the simulation and do not transition further. Nonadherence disqualifies a patient from experiencing either pain relief or adverse events from a given agent. Serious or intolerable adverse events trigger discontinuation of current therapy (with or without adjunctive cannabis) and drug-switching. Patients who attain good pain relief (pain score <4) transition to the mild pain state at the end of the cycle. In the absence of good pain relief, patients remain in moderate-to-severe pain at the end of the cycle. SAE, serious adverse events.

All patients began the simulation in the moderate-to-severe pain state (i.e., score ≥4 on an 11-point Likert scale). Patients were assessed stepwise for mortality, adherence, adverse events, and pain relief following procedures developed by Bellows et al.^31^ modified to a 6-week cycle to account for additional pain relief and/or adverse events related to adjunctive cannabis use (Fig. 1). Six-week cycle length was selected as this represents a patient with moderate-to-severe pain whose treatment is actively being optimized.

At the end of each 6-week cycle, patients with a pain score <4 were assumed to have good pain relief and improved quality of life. If so, patients would remain on that treatment. Patients with a pain score ≥4 had poor pain relief and decreased quality of life. If patients had two or more 6-week cycles of inadequate pain control or experienced intolerable or serious adverse events (SAEs) in any one cycle, it was assumed that they would switch drugs. Patients who were nonadherent to adjunctive cannabis experienced poor pain relief through two cycles of adjunctive cannabis treatment, or experienced intolerable or SAEs while taking adjunctive cannabis, were considered to have failed cannabis and could not restart the drug.

### Model inputs

Parameters selected for the model, model inputs, and their distribution types are presented in Table 1. Model inputs for the efficacy and adverse event rates of standard therapy agents were extracted from Bellows et al.^31^ Costs were assessed from a U.S. health care sector perspective, which incorporates both third-party payer direct medical costs (i.e., what insurers pay) and patient out-of-pocket costs, to account for both health plan-covered prescription medications and medicinal cannabis, which patients must pay for themselves.^24^ The cost of standard therapy agents, health state utility, and utility decrements due to adverse events were adapted from Bellows et al. (Supplementary Table S1).^31^

**Table 1.** Parameter Distribution Inputs

Parameter	Model inputs	Distribution type
Baseline values,^31^ mean (SD)		
Age	59.72 (9.79)	Normal
Pain score	6.20 (1.52)	Normal
Pain score reduction,^31^ mean (SD)		
Duloxetine	2.57 (2.31)	Normal
Desipramine	1.99 (2.16)	Normal
Gabapentin	2.42 (2.34)	Normal
Pregabalin^31^	2.59 (1.87)	Normal
Cannabis^35^	1.11 (2.38)	Normal
Probability of nonserious AEs,^31^ proportion (SE)		
Duloxetine	66.0% (1.2%)	Beta
Desipramine	74.4% (4.9%)	Beta
Gabapentin	66.4% (2.5%)	Beta
Pregabalin	69.1% (1.5%)	Beta
Cannabis^35,a^	58.6% (3.4%)	Beta
Probability of intolerable AEs,^31^ proportion (SE)		
Duloxetine	15.7% (1.2%)	Beta
Desipramine	13.8% (4.5%)	Beta
Gabapentin	14.7% (2.3%)	Beta
Pregabalin	12.5% (1.3%)	Beta
Cannabis^35,a^	4.6% (1.4%)	Beta
Probability of serious AEs,^31^ proportion (SE)		
Duloxetine	2.4% (0.4%)	Beta
Desipramine	1.3% (1.3%)	Beta
Gabapentin	4.0% (1.1%)	Beta
Pregabalin	2.6% (0.5%)	Beta
Cannabis^35,a^	0.5% (0.5%)	Beta
Cannabis AE risk modifier,^35^ odds ratio (95% CI)		
Nonserious AEs	1.74 (1.42–2.14^b^)	Logistic
Nonserious AEs—no active use	2.07 (1.59–2.70^b^)	Logistic
Serious AEs	1.08 (0.57–2.04^b^)	Logistic
Serious AEs—no active use	1.77 (0.72–4.32^b^)	Logistic
Risk of death from SAE^59^ (by age, years), proportion (SE)		
18–44	1.2% (0.1%)	Beta
45–64	1.6% (0.2%)	Beta
65–84	1.9% (0.2%)	Beta
≥85	2.6% (0.6%)	Beta
Adherence,^60^ mean (SD)		
Duloxetine	0.86 (0.18)	Beta
Desipramine	0.76 (0.24)	Beta
Gabapentin	0.74 (0.24)	Beta
Pregabalin	0.69 (0.25)	Beta
Cannabis^36^	0.84 (95% CI: 0.78–0.90^b^)	Beta
Adherence threshold^c^ (assumed)	0.8 (range: 0.5–1.0)	Triangular
Discontinuation rate,^31^ proportion (SE)		
Duloxetine	1.7% (0.4%)	Beta
Desipramine	2.6% (1.8%)	Beta
Gabapentin	2.3% (0.8%)	Beta
Pregabalin	3.9% (0.7%)	Beta
Cannabis^35^	10.7% (2.1%)	Beta
Health state utilities,^13^ mean (SD)		
Mild pain	0.7 (0.2)	Beta
Moderate-to-severe pain	0.39 (0.33)	Beta
Utility decrements, mean		
Tolerable AE^61,62^	0.05^d^	Beta
Intolerable AE^63–65^	0.11^d^	Beta
Serious AE^64,66,67^	0.12^d^	Beta
Office visit costs,^68^ mean (SD)		
Regular visit	$111 ($7)	Gamma
SAE visit	$150 ($10)	Gamma
Regular visit, out-of-pocket^69^	$51 ($4)	Gamma
SAE visit, out-of-pocket^69^	$57 ($8)	Gamma
SAE hospitalization costs^59^ (by age, years), mean (SE)		
18–44	$7,387 ($130)	Gamma
45–64	$9,447 ($165)	Gamma
65–84	$9,664 ($292)	Gamma
≥85	$8,658 ($340)	Gamma
Hospitalization out-of-pocket costs^70^	$70 ($37)	Gamma
Standard therapy wholesale costs^31,71^ (1 month supply), mean (SD)		
Duloxetine	$254 ($20)	Gamma
Desipramine	$236 ($58)	Gamma
Gabapentin	$305 ($99)	Gamma
Pregabalin	$485 ($33)	Gamma
Standard therapy out-of-pocket costs,^71,72^ mean (SE)		
Duloxetine	$13.00 ($2.34)	Gamma
Desipramine	$22.25 ($7.04)	Gamma
Gabapentin	$8.79 ($3.54)	Gamma
Pregabalin	$19.63 ($9.98)	Gamma
Cannabis cost, mean (SD)		
Price per gram^37^	$11.06 ($3.78)	Gamma
Cannabis quantification^6^		
Daily grams THC	0.067 (0.034)	Gamma
Cannabis wastage	38.9% (13.2%)	Beta

^a^ For cannabis “monotherapy,” when patient is nonadherent to conventional agent but adherent to cannabis.

^b^ Distribution SD estimated as 1/4 of 95% CI.

^c^ Range and distribution for adherence threshold used in probabilistic sensitivity analysis only.

^d^ Distribution SD estimated as 1/2 of mean value.

AE, adverse event; CI, confidence interval; SAE, serious adverse event; SD, standard deviation; SE, standard error; THC, tetrahydrocannabinol.

To derive parameters for efficacy of smoked cannabis, we examined clinical trials of whole-plant cannabis in adults with chronic peripheral neuropathic pain if the study drug was administered in cigarette form, 24-h average pain scores were reported, pain reduction was reported on a numeric or Likert scale, duration was ≥5 days, and results were published in English. We considered all etiologies for neuropathic pain. Two trials in HIV-associated sensory neuropathy were identified which met our criteria.^6,7^ However, we required access to study data to extract mean pain score reductions. Only Ellis et al. provided data for their study. Data from the other trial were not easily accessible (Donald Abrams, personal communication).

We converted the pain score reduction measures from a 100-point visual analog scale to an 11-point Likert scale to align with the parameters for standard therapy agents. We modeled the efficacy of adjunctive cannabis using the mean difference in pain score reductions between active cannabis and placebo cigarettes.

To model the change in probability of adverse events when cannabis is used to augment standard therapy, we applied a modifier to adverse event rates for standard therapy agents. This modifier was derived from adjusted odds ratios (ORs) for non-SAE and SAE calculated in the Cannabis for the Management of Pain: Assessment of Safety Study (COMPASS),^35^ which compared safety outcomes between chronic pain patients who did and did not self-treat with cannabis over a 1-year timeframe. When patients were nonadherent to standard therapy but adherent to cannabis, adverse event rates were derived from the proportion of participants in COMPASS who were exposed to cannabis and experienced adverse events that investigators considered related to the drug.

Dosing of cannabis was based on the administration schedule used by Ellis et al., that is, four times daily.^6^ Literature on adherence to medicinal cannabis is sparse, and no published study reports cannabis adherence as a scalar variable. Therefore, we estimated adherence to smoking cannabis four times daily as adherence to a four times daily-dosed medication in chronic disease, reported in a recent meta-analysis.^36^

We derived the cost per gram of cannabis flower from a study of transactions in the Washington state legal marketplace from 2014 to 2016.^37^ We modeled a cannabis whole-plant product containing 12.5% tetrahydrocannabinol (THC), which emulates the study cannabis used in COMPASS.^35^ The daily dose of THC was derived from the average daily dose delivered to participants by Ellis et al. in their clinical trial,^6^ a dose individually titrated to balance pain relief and tolerability.

### Analysis

Effectiveness of each treatment strategy was expressed in QALYs. In the model, QALYs for each 6-week cycle were calculated by multiplying the utility of a patient's health state, determined by pain relief and adverse events, by the time spent in that health state.^30^ Utility, a measure of quality of life assigns a value between 0 (death) and 1 (perfect health) to represent severity of disability in a health state. Costs were also assessed on a 6-week cycle. Costs included both third-party and out-of-pocket costs due to the standard therapy agents, costs of an office visit to a physician or hospitalization, and, where applicable, costs of adjunctive cannabis. The cost-effectiveness of treatment arms was expressed using ICERs. We adjusted costs and QALYs at a rate 3% annually to account for inflation and adjusted future utility gains to their value at present.^24^ All analyses were performed using TreeAge Pro 2018 (TreeAge Software, Inc., Williamstown, MA).

#### Base-case analysis

The goals of our analysis were twofold. First, we evaluated whether any adjunctive cannabis strategy was cost-effective compared to usual care by determining if the calculated ICER was at or below a willingness-to-pay threshold of $100,000 per QALY. Second, we compared the relative cost-effectiveness of first-line, second-line, and third-line adjunctive cannabis to determine the strategy associated with the greatest value.

#### Sensitivity analysis

To assess the robustness of our findings to variations in model parameters—as some parameters may fluctuate in clinical practice—we performed both one-way and probabilistic sensitivity analyses. This is especially relevant to medicinal cannabis for which prices fluctuate widely by region and retailer and for which there remains uncertainty regarding long-term efficacy and rates of adverse events.^37–40^

In one-way sensitivity analysis, the model is run for multiple iterations and the mean of a single parameter is varied over a specified range. For cannabis adverse event rate modifiers and adherence to adjunctive cannabis, we defined this range as the 95% confidence interval of reported ORs. Mild pain health state utility, cannabis price per gram, daily THC dose, and adherence to standard therapy agents were all varied over ±one standard deviation from the mean. Adherence threshold was varied from 0.5 to 1.0 (i.e., 50% adherence to 100% adherence). Non-SAE probabilities were varied over ±25% from the mean. All other parameters were varied over ±50% from the mean value.

We also performed probabilistic sensitivity analysis to account for uncertainty across all parameters at once. The model was run 10,000 times with new parameter values selected at each iteration from the model distributions used in base-case analysis (Table 1). While adherence threshold was static in base-case analysis, in probabilistic sensitivity analysis, it was sampled from a triangular distribution from 0.5 to 1.0. The resulting QALYs and costs were used to determine the relative cost-effectiveness of each treatment plan. The proportion of model iterations, in which a given treatment plan was most cost-effective compared to all other treatment arms, was plotted against a range of willingness-to-play thresholds, the cost-per-QALY value up to which interventions are considered cost-effective in a given context.

#### Alternate time horizons

In base-case, our model was analyzed with a 1-year time horizon. This was a function of limited data in the literature: the longest timeframe, in which adverse events were followed for users of a medicinal cannabis regimen, was 1 year.^35^ As use of an analgesic regimen beyond 1 year is more reflective of clinical practice for a chronic condition such as painful neuropathy, we examined the model with extended time horizons of 5 and 10 years. However, these estimates were highly exploratory as data on the efficacy and adverse effects of medicinal cannabis use in that timeframe are unavailable.

#### Alternate adverse event modifiers

Adverse event rate modifiers for cannabis-containing regimens were derived from adjusted ORs calculated in an observational cohort study, in which 65.6% of participants in the cannabis-exposed group were current users at baseline.^35^ Tolerance to adverse effects has been observed after repeated dosing.^41^ ORs for all adverse events and SAEs were higher when active cannabis users were excluded from analysis. We conducted a subanalysis using these ORs to simulate starting medicinal cannabis in a population who are not active users.

#### Cannabis wastage

The base-case model assumes that patients use cannabis with a high degree of efficiency, consuming only the amount corresponding to their prescribed dose of THC. To simulate loss of cannabis to waste (and the need to purchase a somewhat larger quantity of the drug than in base-case), we calculated a cannabis wastage term using supplemental data provided by Ellis et al.^6^—defined as the proportion of unused cannabis cigarette at the end of a smoking session multiplied by THC concentration—and applied it to our model.

## Results

### Base-case analysis

The results of the base-case analysis are presented in Table 2. In the base-case analysis, usual care had the lowest mean cost ($6,397) per patient, followed by third-line ($6,641), second-line ($7,007), and first-line adjunctive cannabis strategies ($7,234). Second-line adjunctive cannabis provided the greatest average QALYs per patient (0.489), followed by first-line (0.488) and third-line (0.480) adjunctive cannabis, then usual care (0.476).

**Table 2.** Average Cost and Efficacy of Base-Case Analysis

Treatment strategy	Average		Incremental		ICER ($/QALY gained)
	Cost ($U.S.)	Efficacy (QALY)	Cost	Efficacy	
Usual care	$6,397	0.476	REF	REF	REF
First-line adjunctive cannabis	$7,234	0.488	—	—	Dominated
Second-line adjunctive cannabis	$7,007	0.489	$610	0.013	$48,594
Third-line adjunctive cannabis	$6,641	0.480	—	—	Ext. dominated

All costs are in 2017 U.S. dollars. ICERs are calculated referent to the next least costly nondominated treatment option.

ICER, incremental cost-effectiveness ratio; REF, reference value; Ext. dominated, extendedly dominated; QALY, quality-adjusted life-years.

As it costs more while being less effective than second-line adjunctive cannabis, first-line adjunctive cannabis was dominated and not considered further. While both second- and third-line adjunctive cannabis strategies were more effective than usual care, second-line adjunctive cannabis yielded more QALYs and had a more favorable ICER versus usual care; thus, third-line adjunctive cannabis was extendedly dominated and not considered further. Second-line adjunctive cannabis was cost-effective compared to usual care, with an ICER of $48,594 per QALY gained.

### Sensitivity analyses

**FIG. 2.**
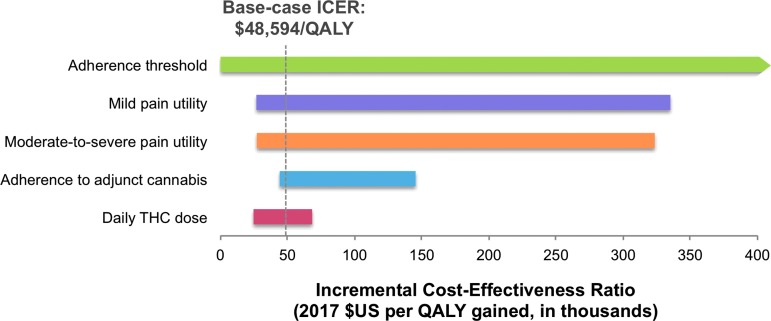
One-way sensitivity analysis tornado diagram. ICER represents the incremental cost per QALY gained from second-line adjunctive cannabis when compared to usual care. The dotted vertical line represents the base-case ICER of $48,594/QALY, while the horizontal bars indicate the magnitude of change in ICER caused by varying the parameter over its specified range. A negative ICER value, at which second-line adjunctive cannabis was dominated by usual care, is represented by an arrow tip on the end of the horizontal bar. All variables were examined in analysis; the five parameters shown caused the greatest change in ICER. Varying adherence threshold caused usual care to dominate second-line adjunctive cannabis. Varying moderate-to-severe pain state utility, mild pain state utility, and adherence to adjunctive cannabis over their respective ranges for sensitivity analysis caused the ICER to cross the $100,000/QALY threshold and second-line adjunctive cannabis to lose cost-effectiveness. However, second-line adjunctive cannabis remained cost-effective across the range of values for daily THC dose inputs. ICER, incremental cost-effectiveness ratio; QALY, quality-adjusted life-year; THC, tetrahydrocannabinol.

As second-line adjunctive cannabis dominated or extendedly dominated both first-line and third-line strategies in base-case analysis, we structured one-way sensitivity analyses comparing second-line cannabis with usual care (Fig. 2). Our model was most sensitive to changes in adherence threshold, mild pain state utility, and moderate-to-severe pain state utility. The model was also sensitive—to a lesser extent—to changes in cannabis adherence and daily THC dose. For example, when cannabis adherence was reduced from 84% in base-case to 78%, the ICER for second-line adjunctive cannabis tripled to $145,292 per QALY. When daily THC dose required was raised from 0.067 to 0.101 g per day, the ICER for second-line cannabis increased to $68,220 per QALY.

**FIG. 3.**
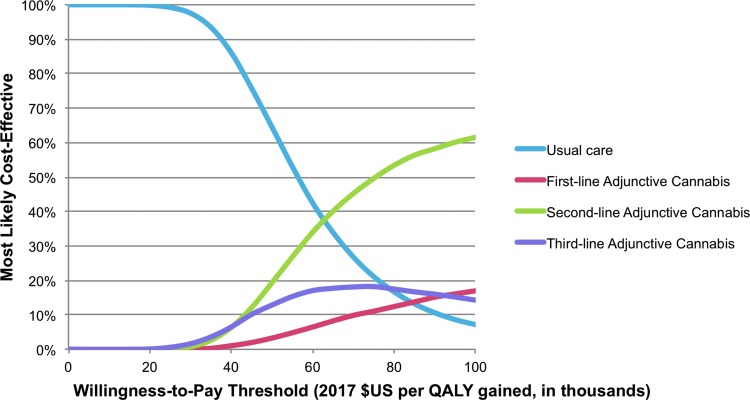
Cost-effectiveness acceptability curve from probabilistic sensitivity analysis. Percentage of iterations, in which a given treatment strategy is most cost-effective, was plotted against escalating willingness-to-pay thresholds. In our model, cannabis-containing strategies were most cost-effective at willingness-to-pay thresholds of approximately $60,000 per QALY gained and above.

In probabilistic sensitivity analysis, usual care was most likely to be cost-effective up to a willingness-to-pay threshold of $60,000 per QALY (Fig. 3). Above $60,000 per QALY second-line adjunctive cannabis was the most cost-effective strategy, with a 62% probability of being the most cost-effective strategy at a willingness-to-pay threshold of $100,000 per QALY.

### Alternate time horizons

Adjunctive cannabis remained cost-effective in analyses using time horizons of 5 and 10 years (Table 3). First-line adjunctive cannabis was dominated in all alternate timeframes, while third-line adjunctive cannabis was no longer extendedly dominated by second-line adjunctive cannabis, with more favorable ICERs over both 5- and 10-year time horizons.

**Table 3.** Outcomes of Alternate Time Horizon, Adverse Event Rate, and Cannabis Wastage Analyses

	Usual care	First-line adjunctive cannabis	Second-line adjunctive cannabis	Third-line adjunctive cannabis
Five-year time horizon				
Average QALYs	2.024	2.057	2.098	2.088
Average cost	$27,505	$29,035	$29,327	$28,865
ICER	REF	Dominated	$45,968	$21,143
Ten-year time horizon				
Average QALYs	3.469	3.507	3.563	3.557
Average cost	$47,937	$49,926	$50,333	$49,853
ICER	REF	Dominated	$81,591	$21,834
Alternate cannabis AE rates				
Average QALYs	0.476	0.482	0.486	0.479
Average cost	$6,379	$7,643	$7,167	$6,688
ICER	REF	Dominated	$73,193	Ext. dominated
Cannabis wastage				
Average QALYs	0.476	0.488	0.489	0.480
Average cost	$6,383	$7,875	$7,430	$6,797
ICER	REF	Dominated	$83,865	Ext. dominated

All costs are in 2017 U.S. dollars. ICERs are calculated referent to the next least costly nondominated treatment option.

### Alternate adverse event rate modifiers

When the adverse event rate modifiers for cannabis were adjusted to reflect the exclusion of active cannabis users from the patient cohort, second-line adjunctive cannabis continued to dominate other cannabis-containing strategies (Table 3). The strategy produced a slightly lower increase in efficacy from base-case (0.010 from 0.013), with a slight increase in incremental cost ($788 from $610), which resulted in an ICER of $73,193 per QALY.

### Cannabis wastage

Results of cannabis wastage subanalysis are presented in Table 3. As in base-case analysis, second-line adjunctive cannabis dominated first-line and third-line adjunctive cannabis. Applying cannabis wastage to the base-case model resulted in a significantly higher ICER ($83,865 per QALY).

## Discussion

In this exploratory CEA of smoked cannabis for neuropathic pain, we found augmentation of standard therapy agents for neuropathic pain with smoked cannabis to be cost-effective over the short- and long-term with ICERs below our designated threshold of $100,000 per QALY. This held true when parameters were varied over their distribution ranges in probabilistic sensitivity analysis, and when more stringent inputs for adverse event rates and cannabis wastage were modeled.

First-line adjunctive cannabis was dominated by other adjunctive cannabis strategies, as exposing the entire cohort of patients to the increased risk of non-SAEs associated with cannabis use appeared to outweigh any significant gains in pain control. In contrast, when patients started cannabis after failing one or more drugs—through either repeated rounds of poor pain relief or intolerable adverse events—they derived increased utility at a more favorable cost. In the base-case analysis, this was most apparent for second-line adjunctive cannabis.

No published CEA has addressed the use of smoked cannabis, the treatment of neuropathic pain, or cannabis and cannabinoids in the United States health marketplace; our study, then, is a novel contribution to the literature in all three regards. Previous studies have evaluated the cost-effectiveness of cannabis-based medicine for the treatment of MS in the European health marketplace, with conflicting results. One combined randomized clinical trial and CEA of dronabinol to slow disease progression found that the intervention had no significant disease-modifying effect and was therefore not cost-effective.^42^ Four published CEAs evaluated nabiximols versus usual care for the treatment of MS-related spasticity using a willingness-to-pay threshold of €30,000 per QALY or £30,000 per QALY. A publicly funded study in the United Kingdom found that nabiximols were not cost-effective at that threshold, while industry-funded studies built using Spain, Germany, Italy, and Wales as health marketplace settings found nabiximols to be cost-effective.^43–46^

Our model was sensitive to changes affecting the quantity of cannabis purchased and, therefore, the price paid for cannabis. We overestimated quantities of cannabis consumed daily: dosing of THC was based on the weight of cannabis cigarette consumed by participants in the clinical trial by Ellis et al.,^6^ but it was impossible to subtract the weight of the paper used to roll the cigarette. Likewise, the per-gram price figure used in their analysis may have overestimated cost, as the majority of transactions used to calculate it were subject to a 37% excise tax not levied on medicinal cannabis.^37,47^ In practice, patients in the United States pay a variety of prices, which may be much higher in some settings or significantly lower, especially if patients grow their own cannabis.

Our model incorporates parameters from a single clinical trial of good quality, a year-long observational study, and published market data to simulate the use of cannabis in clinical practice as comprehensively as possible. Nonetheless, our model has several limitations. Existing clinical trials of cannabis have been of short duration, we overestimated the dose of cannabis consumed because the weight of the paper used to roll the cigarettes was included in measurements of the dose, and the observational cohort study may under- or overestimate adverse event rates related to long-term use of cannabis.

Due to the limited scope of published clinical trials of cannabis and the absence of CEA in HIV neuropathy, we derived cannabis efficacy parameters from participants with HIV neuropathy and then applied them to a published CEA model of pDPN. However, there is evidence in the literature to suggest that this is a reasonable extrapolation: pain relief from standard therapy agents is similar in HIV neuropathy and pDPN^48–53^; treatment guidelines for both pDPN and HIV neuropathy recommend standard therapy agents as first- or second-line agents^54–56^; short-term effects of smoked cannabis for pDPN are consistent with effects observed in trials on HIV neuropathy.^6–8,11^

Another limitation is that there are no studies of inhaled medical cannabis examining “tolerance” to cannabis over the long-term, and tolerance was not accounted for in the model. However, a study of cannabinoids in MS suggest continued analgesic effects over time.^57^ Furthermore, our model inputs for adverse events were derived from an observational cohort, in which patients administered their study cannabis by multiple routes, including smoking, vaporization, and oral consumption (the majority of patients—61%—using some combination of the three^35^). As such, they may not be generalizable to an exclusively smoked cannabis regimen.

Because there are no available clinical trial data on long-term consequences of regular licit, smoked medicinal cannabis use—with data on long-term outcomes derived from studies of recreational or other illicit use^58^—we did not structure provisions for latent, insidious or long-term adverse effects into the model.

## Conclusion

Notwithstanding limitations, this is the first published CEA of inhaled cannabis for any condition. The results of our analysis indicate that should long-term consequences and efficacy be similar to what has been observed in published trials, smoked medicinal cannabis is a useful tool from a cost-effectiveness perspective for the treatment of chronic neuropathic pain. Judicious use of medicinal cannabis alongside standard therapy agents may be particularly beneficial to patients with refractory pain and to active cannabis users. Our findings are concordant with clinical experience and published guidelines that recommend consideration of cannabis for patients nonresponsive to initial treatment.^16^

The data demonstrate the importance of cost to the ultimate utility of medicinal cannabis in practice. In addition to exploring the efficacy and safety profile of cannabis-based medicine, including cannabis or cannabinoids as monotherapy for neuropathic pain, future research should evaluate its economic feasibility and influence public policy to assure that this potentially useful intervention is accessible. Characterizing the cost-effectiveness of medicinal cannabis will inform future research and policy as to whether this treatment modality is promising from a health economics and population health perspective, over and above its emerging efficacy in clinical trials.

## Supplementary Material

Supplemental data

## Abbreviations Used

AE: adverse event
COMPASS: Cannabis for the Management of Pain: Assessment of Safety Study
CI: confidence interval
CEA: cost-effectiveness analysis
Ext. dominated: extendedly dominated
ICER: incremental cost-effectiveness ratio
MS: multiple sclerosis
ORs: odds ratios
pDPN: painful diabetic peripheral neuropathy
QALYs: quality-adjusted life years
REF: reference value
SD: standard deviation
SE: standard error
SAEs: serious adverse events
THC: tetrahydrocannabinol
