# Supplementary material for: A Cost-Effectiveness Model for Adjunctive Smoked Cannabis in the Treatment of Chronic Neuropathic Pain
Source: Cannabis Cannabinoid Res. 2019 Mar 13;4(1):62–72. doi: 10.1089/can.2018.0027 (PMC6446169; doi:10.1089/can.2018.0027)
Supplement: Supplemental data [file Supp_Data.pdf]

Supplementary Data

Supplementary Table S1. Additional Model Inputs

Input type	Description	Source
Standard therapy agents		
Monthly third-party cost of prescription desipramine, duloxetine, gabapentin, or pregabalin	Average wholesale price of a 30-day supply of despiramine (150 mg/day), duloxetine (60 mg/day), gabapentin (2400 mg/day), or pregabalin (300 mg/day)	Bellows et al.[S1]
Monthly out-of-pocket cost of prescription desipramine, duloxetine, gabapentin, or pregabalin	Mean out-of-pocket cost for a 30-day supply of tricyclic antidepressant, duloxetine (60 mg/day), gabapentin (2400 mg/day), or pregabalin (300 mg/day)	Medical Expenditures Panel Survey[S2]
Heath service costs		
Regular office visit with physician	25-min long appointment (CPT 99214)	Centers for Medicare and Medicaid Services[S3]
Office visit with physician because of SAE	40-min long appointment (CPT 99215)	Centers for Medicare and Medicaid Services[S3]
Out-of-pocket expenses due to regular office visit with physician	Mean cost of all regular outpatient visits	Medical Expenditures Panel Survey[S4]
Out-of-pocket expenses due to office visit with physician because of SAE	Mean cost of outpatient visits featuring an EKG and/or laboratory test	Medical Expenditures Panel Survey[S4]
Hospitalization due to an SAE	Mean costs, stratified by age group, for inpatient admissions associated with drug and medication adverse events (CCS 242)	Health care Cost and Utilization Project[S5]
Out-of-pocket expenses due to hospitalization because of SAE	Mean cost of inpatient admissions associated with drug and medication adverse events (CCS 242)	Medical Expenditures Panel Survey[S6]
Background mortality		
Baseline mortality	Mortality based on age from vital statistics tables for 2014	Centers for Disease Control and Prevention[S7]
Mortality related to SAE	Proportion of patients who died during a hospital admission for drug and medication adverse events (CCS 242), stratified by age group	Health care Cost and Utilization Project[S5]
Health utility decrements related to adverse events		
Tolerable adverse event	Reference patient with pDPN who experiences dry mouth	Bellows et al.[S1]
Intolerable adverse event	Reference patient with pDPN who experiences nausea and vomiting	Bellows et al.[S1]
SAE	Reference patient with pDPN who experiences a myocardial infarction	Bellows et al.[S1]

Model inputs related to the cost of standard therapy agents, health service costs, mortality, and health utility, and decrements were adapted from Bellows et al.[S1] All cost figures were updated for 2017 or, where necessary, inflated to 2017 U.S. dollars using the Consumer Price Index available from the Bureau of Labor Statistics.[S8]

CCS, Clinical Classification Software code; CPT, Current Procedural Terminology code; pDPN, painful diabetic peripheral neuropathy; SAE, serious adverse event.

Supplementary References

S1. Bellows BK, Nelson RE, Oderda GM, et al. Long-term cost-effectiveness of initiating treatment for painful diabetic neuropathy with pregabalin, duloxetine, gabapentin, or desipramine. Pain. 2016;157:203–213.

S2. Medical Expenditure Panel Survey (MEPS). Prescribed medicines, 2015. Agency for Healthcare Research and Quality. Available at: www.ahrq.gov/data/meps.html (last accessed on March 23, 2018).

S3. CMS. Physician fee schedule. Centers for Medicare & Medicaid services. Available at: https://www.cms.gov/apps/physician-fee-schedule/ (last accessed on February 5, 2018).

S4. Medical Expenditure Panel Survey (MEPS). Outpatient visits, 2015. Agency for Healthcare Research and Quality. Available at: www.ahrq.gov/data/meps.html (last accessed on March 23, 2018).

S5. HCUPnet—Hospital Inpatient National Statistics. Healthcare cost and utilization project. Available at: https://hcupnet.ahrq.gov (last accessed on February 5, 2018).

S6. Medical Expenditure Panel Survey (MEPS). Hospital inpatient stays, 2015. Agency for Healthcare Research and Quality. Available at: www.ahrq.gov/data/meps.html (last accessed on March 23, 2018).

S7. Arias E, Heron M, Xu J. United States life tables, 2014. Natl Vital Stat Rep. 2017;66:1–64.

S8. Consumer Price Index Inflation Calculator. Bureau of Labor Statistics. Available at: https://data.bls.gov/cgi-bin/cpicalc.pl (last accessed on February 5, 2018).
